# Are women in Uganda gaining adequate gestational weight? A prospective study in low income urban Kampala

**DOI:** 10.1186/s12978-018-0608-2

**Published:** 2018-09-24

**Authors:** Ronald Wanyama, Gerald Obai, Pancras Odongo, Mike N. Kagawa, Rhona K. Baingana

**Affiliations:** 1 Lecturer, Biochemistry Department, Faculty of Medicine, Gulu University, P.O. Box 166, Gulu, Uganda; 2 Lecturer, Physiology Department, Faculty of Medicine, Gulu University, P.O. Box 166, Gulu, Uganda; 3 Lecturer, Internal Medicine Department, Faculty of Medicine, Gulu University, P.O. Box 166, Gulu, Uganda; 4 Lecturer, Obstetrics & Gynecology Department, College of Health Sciences, Makerere University, P.O. Box 7062, Kampala, Uganda; 5 Lecturer, Biochemistry and Sports Science Department, School of Biological Sciences, Makerere University, P.O. Box 7062, Kampala, Uganda

**Keywords:** Pregnancy, Body mass index, Gestational weight gain, Maternal, Uganda

## Abstract

**Background:**

Pre-pregnancy weight and weight gained during pregnancy significantly influence maternal and infant health. Little information is available regarding optimal gestational weight gain (GWG) in relation to pre-pregnancy body mass index (BMI) in Uganda. The study aimed at determining gestational weight gain (GWG) in women pregnant for the first and second time.

**Methods:**

The study was prospective cohort study which included 221 HIV negative women pregnant for the first or second time. It was conducted in the antenatal clinic of the directorate of gynecology and obstetrics, Mulago hospital and women were recruited at ≤18 weeks of gestation by dates. Follow up measurements were done at 26 and 36 weeks gestation. Measured maternal height and reported pre-pregnancy weight were used to calculate BMI. Depending on BMI category, GWG was categorized as inadequate, adequate and excessive based on the Uganda Ministry of Health guidelines.

**Results:**

The participants’ mean ± standard deviation (Sd) age was 20.9 ± 2.7 years and mean ± Sd BMI was 21.40 ± 2.73 kg/m^2^. None of the participants was obese and 68.8% (*n* = 132) were pregnant for the first time. The mean ± Sd GWG at time of delivery was 10.58 ± 2.44 kg. Inadequate GWG was recorded in 62.5% (*n* = 120/192) while only 3.1% (*n* = 6/192) of the participants gained excessive weight during pregnancy.

**Conclusion:**

About 62% of pregnant women in Kampala did not gain adequate weight during their first/second pregnancy. We recommend that studies be carried out to assess whether the Uganda Ministry of Health recommendations for weight gain during are appropriate for preventing adverse pregnancy outcomes across populations in Uganda.

## Plain English summary

Gaining excessive or inadequate weight by women during pregnancy is associated with many adverse maternal and fetal outcomes, either at short or long term. In Uganda, little information is available regarding the weight gained by women during pregnancy. The study aimed at determining gestational weight gain (GWG) in women pregnant for the first and second time.

We conducted a one year prospective study at the antenatal clinic of the directorate of obstetrics and gynecology, Mulago hospital, a national referral public hospital in Uganda. We enrolled 221 women who were pregnant for first or second time as they registered at the clinic. Body mass index (BMI) was calculated from the measured height at recruitment and the reported pre-pregnancy weight. We did follow up measurements at 26 and 36 weeks gestation.

The average age and BMI were 20.9 years and 21.4 kg/m^2^ respectively. The average weight gained at time of delivery was 10.58 kg. Women who gained less weight than needed were 62.5% and those who gained more than needed were 3.1% based on the Uganda Ministry of Health recommendations.

In conclusion, in Uganda, more than 62% of the women in low income urban Kampala do not gain enough weight in pregnant. Women who are overweight prior to getting pregnant have higher chance of gaining enough weight in pregnancy. Therefore, there is a need to assess whether the Uganda Ministry of Health recommendations for weight gain during are appropriate for preventing adverse pregnancy outcomes across populations in Uganda.

## Background

Pregnancy is a critical stage of development during which maternal nutrition strongly influence obstetric and neonatal outcomes [1]. Optimal nutrition is necessary to maintain the health of the mother, to help ensure a normal, healthy delivery, and also to reduce the risk of birth defects, sub-optimal fetal development and chronic health problems in childhood [2]. In Uganda, just as in other developing countries, maternal undernutrition and inadequate gestational weight gain (GWG) are very common [3–5]. The prevalence of obesity or overweight in pregnancy is also rising and is of international concern [6]. Both excessive and inadequate GWG are associated with many adverse maternal and fetal outcomes, either at short or long term [3, 7, 8]. The adverse outcomes include preterm birth, fetal deaths, gestational diabetes, low birth weight and intrauterine growth restriction or small for gestational age babies, pre-eclampsia and complicated deliveries among others. If a woman gains excessive gestational weight and fails to return to her pre-pregnancy weight after delivery, the retained weight has long term health effects on a woman and also adds a burden to future health care cost in the society [9].

The Uganda Ministry of Health (MoH) adapted the 2009 Institute of Medicine (IOM) recommendations and currently recommends GWG per BMI pre-pregnancy category as shown in Table 1 below [4]. Ideally, this recommendation restores maternal fat stores in underweight women while minimizing fat gain in obese women. There are three classifications of GWG according to IOM guidelines; these are inadequate, adequate and excessive GWG.

**Table 1:** Uganda MoH recommendations for total and rate of weight gain during pregnancy, by pre-pregnancy BMI

Pre-pregnancy BMI	BMI (kg/m^2^)	Total weight gain range (kg)	Rates of weight gain 2nd and 3rd trimester (mean range in kg/week)
Underweight	< 18.5	12.5–18	0.51 (0.44–0.58)
Normal weight	18.5–24.9	11.5–16	0.42 (0.35–0.50)
Overweight	25.0–29.9	7–11.5	0.28 (0.23–0.33)
Obese	≥ 30.0	5–9	0.22 (0.17–0.27)

Source: Guidelines on Maternal Nutrition in Uganda (2010) adopted from Institute of Medicine, 2009

In developing countries, inadequate GWG is more prevalence than adequate GWG in women [3, 10, 11] and the reverse is true in developed countries [9, 12, 13]. For example, a recent report from Nigeria indicated than over 96% of the pregnant women failed to gain adequate gestational weight [10] while a study in Iran found 45.9% of women failing to gain adequate weight in pregnancy [14]. In developing countries, a bigger proportion of pregnant women do not receive advice from health professionals about appropriate GWG [15]. Health professionals’ advice about specific or range of weight gain may be limited by their lack of awareness of the well-documented guidelines [16]. There is little information is available regarding GWG in Uganda and our study aimed at determining the proportion of women in low income urban Uganda who gain the needed, less needed or more of the needed weigh in pregnancy. We hope that this finding is going to be used by health workers and policy makers to address the issue of inadequate GWG in Uganda.

## Methods

### Study design, site and population

This was a one year prospective study conducted between May 2013 and May 2014 at the antenatal clinic of the directorate of Obstetrics and Gynecology, Mulago national referral hospital (formerly Kawempe health centre IV). The Hospital is supported by the Uganda Ministry of Health and the services provided are free to the public. The Hospital serves mainly low-income population in Kampala and Wakiso Districts in Uganda. The study participants were women who were pregnant for the first or second time. We used online openEpi software, http://www.openepi.com, based on Kelsey Lesley (1996) to calculate sample size. In the formula we used a confidence level of 95%, power of 80%.

### Enrollment and follow up

The study employed consecutive sampling method where pregnant women who met the inclusion criteria were selected. The selection of women was done after they had registered at the antenatal clinic. Following registration, written informed consent was obtained from eash woman who volunteered to participate. The inclusion criteria were; being able to recall her last normal menstrual period, gestation age equal to or less than 18 weeks based on the reported last menstrual period were enrolled, being HIV negative and pregnant for the first or second time, carrying a singleton pregnancy. Furthermore, a woman had to be 18–35 years of age and free of any systemic illness such as active peptic ulcers, hypertension and diabetes mellitus. Nevertheless, some individuals were excluded from based on the following criteria; having a genetic abnormality like sickle cell disease, not able to schedule their return visits, not able to recall their pre-pregnancy weight, history of drug or alcohol abuse and mental illness. Based on the set exclusion criteria, a total of fifty six pregnant women were disqualified from our study. Twenty-eight of them could not recall their pre-pregnancy weight, fourteen could not adhere to the scheduled return visits, six had active peptic ulcers, four had alcohol related problems, two were carrying twin pregnancies (based on ultrasound scan results) and two had sickle cell disease. Anthropometric measurements were made at recruitment, 26 weeks of gestation and 36 weeks of gestation.

### Anthropometric measurements

Anthropometric measurements were performed by a trained measurer in a private room with the help of a midwife. A portable adult beam scale with 150 kg capacity (Gmbh & co.kg, Germany model 7,621,019,009) was calibrated every morning and used to measure weight of participants. A height board consisting of a non-extendable two meter measuring was used to measure height of participants. Height measurements were performed twice on every participant and the mean of the readings was considered as the height. The BMI of each participant was calculated as follows: BMI = pre-pregnancy weight (kg)/height (m) squared. The BMI was categorized using the World Health Organization criteria [17]. Pre-pregnancy weight (*W*_Pre_) considered in this study was that reported by the participant at recruitment. The measured gestation weight at each time point (*GW*_Rec_ = weight at recruitment, *GW*_26_ = weight at 26 weeks of gestation and *GW*_36_ = weight at 36 weeks of gestation) was recorded. Rate of GWG during second trimester was calculated as {(*GW*_26_ – *GW*_Rec_)/(26 – gestation age at recruitment)}kg/week. Similarly, rate of GWG during third trimester was calculated as {(*GW*_36_ – *GW*_26_)/10} kg/week. GWG by 36 weeks was calculated by subtracting *W*_Pre_ from *GW*_*36*_*.* We subtracted 36 weeks from gestation age at delivery in weeks and multiplied with rate of GWG during the third trimester to get the weight gained (*WG*_*d*_) from last measurement at 36 week to delivery; *WG*_*d*_ = {(gestation age at delivery – 36)*rate of GWG during the third trimester}kg. Total GWG was estimated as follows; GWG = {(*GW*_36_ *+ WG*_d_) – *W*_pre_}kg. We specifically aimed at determining the proportion of women who gain adequate, inadequate and excess weight based on pre-pregnancy BMI (as detailed in Table 1) during pregnancy.

### Analysis of data

Statistical Package for Social Sciences (SPSS) V.16.0 was used to analyze the data. Socio-demographic characteristics and anthropometric variables were presented as frequencies and mean ± standard deviation (Sd). The outcome variables of interest were GWG at delivery and the rates of GWG during the second and third trimesters of pregnancy. These two outcome variables were compared to the recommendations by the IOM and level of statistical significance was set at *p* < 0.05.

## Results

A total of 221 women pregnant for the first or second time enrolled and followed until 36 weeks of gestation. Twenty six of the enrolled participants were lost to follow up and two of the participants lost their pregnancies before 36 weeks of gestation and one delivered a preterm baby on the day she was supposed to come for measurement at 36 weeks of gestation. This left us with us with 192 participants to consider for the analysis.

Table 2 shows the socio-demographic characteristics of the participants by GWG. Most of the participants (118/192) were pregnant for the first time. The majority of participants were married (168/192) and (145/192) had no gainful formal employment. Almost all participants were nonsmokers (190/192) and were not taking alcohol (182/192). A good number of participants had attended vocational or tertiary training (150/192) however very few households (11/192) were earning more than 250 US dollars per month.

**Table 2:** Socio-demographic characteristics of the participants by GWG

Variable	Overall total *n* (%)	Less GWG *n* (%)	Adequate GWG *n* (%)	Excess GWG *n* (%)
Parity				
Primigravidae	118 (61.5)	72 (61.0)	42 (35.6)	4 (3.4)
Secundigravidae	74 (38.5)	48 (64.9)	24 (32.4)	2 (2.7)
Occupation				
No formal employment	145 (75.5)	94 (64.8)	48 (33.1)	3 (2.1)
Formal employment	38 (19.8)	23 (60.5)	12 (31.6)	3 (7.9)
Student	9 (4.7)	3 (33.3)	6 (66.7)	0 (0.0)
Marital status				
Married	168 (87.5)	109 (64.9)	53 (31.5)	6 (3.6)
Single	19 (9.9)	10 (52.6)	9 (47.4)	0 (0.0)
Widowed	2 (1.0)	0 (0.0)	2 (100.0)	0 (0.0)
Separated/divorced	3 (1.6)	1 (33.3)	2 (66.7)	0 (0.0)
Smoking				
Yes	2 (1.0)	0 (0.0)	2 (100.0)	0 (0.0)
No	190 (99.0)	120 (63.1)	64 (33.7)	6 (3.2)
Alcohol				
Yes	10 (5.2)	3 (30.0)	7 (70.0.)	1 (10.0)
No	182 (94.8)	118 (64.8)	59 (34.4)	5 (2.8)
Building type				
Permanent	190 (99.0)	119 (62.6)	65 (34.2)	6 (3.2)
Temporary	2 (1.0)	1(50.0)	1 (50.0)	0 (0.0)
Education level				
Low (< secondary)	7 (3.7)	3 (42.9)	3 (42.9)	1 (14.2)
Medium (secondary)	35 (18.2)	23 (65.7)	12 (34.3)	0 (0.0)
High (vocational/tertiary)	150 (78.1)	94 (62.7)	51 (34.0)	5 (3.3)
Household monthly income ($)				
Low income (< 100)	94 (49.0)	59 (62.8)	31 (33.0)	4 (4.2)
Medium income (101–250)	87 (45.3)	51 (58.6)	34 (39.1)	2 (2.3)
High income (> 250)	11(5.7)	10 (90.9)	1 (9.1)	0 (0.0)

*n* number, *GWG* gestational weight gain

Table 3 shows the mean ± Sd of the continous characteristics of participants by pre-pregnancy BMI category. The average age (years) for underweight, normal weight and overweight participants was 19.82, 20.97 and 22.24 respectively. The average weight (kg) for underweight, normal weight and overweight participants was 43.57, 53.16 and 65.14 respectively. The average BMI (kg/m^2^) for underweight, normal weight and overweight participants was 17.65, 21.39 and 26.56 respectively. Here we observe that BMI increased with increasing age and weight of the participants. The average GWG (kg) for underweight, normal weight and overweight participants was 11.31, 10.54 and 9.78 respectively. The average rate of GWG (kg/week) during the second and third trimesters for underweight, normal weight and overweight participants was 0.32, 0.30 and 0.28 respectively. We observe that GWG and rate of GWG decreased with increasing BMI.

**Table 3:** Mean ± Sd of some of the participants’ characteristics by pre-pregnancy BMI category

Variable	Overall Mean ± Sd	Pre-pregnancy BMI (kg/m^2^)		
		Underweight (< 18.5)	Normal weight (18.5–24.9)	Overweight (25.0–29.9)
Age (years)	20.94 ± 2.65	19.82 ± 1.44	20.97 ± 2.49	22.24 ± 4.11
Weight (kg)	53.07 ± 7.61	43.57 ± 3.62	53.16 ± 5.73	65.14 ± 4.79
Height (cm)	157.4 ± 5.77	157.0 ± 5.4	157.6 ± 5.8	156.9 ± 6.3
BMI (kg/m^2^)	21.40 ± 2.73	17.65 ± 0.83	21.39 ± 1.75	26.56 ± 1.28
GWG (kg)	10.58 ± 2.44	11.31 ± 2.98	10.54 ± 2.30	9.78 ± 2.41
RGWG (kg/week)	0.30 ± 0.08	0.32 ± 0.08	0.30 ± 0.08	0.28 ± 0.09

*GWG* gestational weight gain, *RGWG* rate of gestational weight gain during the second and third trimesters

Table 4 shows the numbers of participants who gained less than, adequate or above the recommended GWG and their corresponding mean GWG and mean rates of GWG by BMI categories. Overall, only 34.4% gained the needed weight in pregnancy based on the Uganda MoH recommendation. The majority of the participants, that is, 62.5% gained less than the recommended GWG while only 3.1% gained above the recommended. The mean ± Sd GWG for those who gained less than recommended was 9.34 kg while it was 12.43 kg for those who gained adequate gestational weight. The average GWG for those who gained more than the recommended was 14.83 ± 2.12 kg. By BMI categories, no participant in the underweight category gained gestational weight above the Uganda MoH recommendation. However, two participants from the normal weight category and four participants from the overweight category gained above the Uganda MoH recommendation. Over 70% (20/28) of the underweight participants and 68.5% (98/143) of the normal weight participants gained weight below the Uganda MoH recommendation. However, most of the overweight participants, 71.4% (15/21), gained the weight within the acceptable range. From Table 4 also we observe that underweight participants gained the most weights and had the greatest rates of GWG (kg/week) while overweight participants had the least rates of GWGW. For example, underweight and overweight participants who gained the recommended gestational weight had mean ± Sd values of 15.23 ± 1.10 kg and 9.15 ± 1.20 kg respectively.

**Table 4:** Participants’ mean GWG and rate of GWG during second and third trimesters by pre-pregnancy BMI category according to MoH recommendation

Pre-pregnancy BMI (kg/m^2^)	Number (%)	Mean GWG ± Sd	Mean rate of GWG ± Sd (kg/week)
Underweight (< 18.5)			
Less than recommended GWG	20 (71.4)	9.75 ± 1.76	0.30 ± 0.08
Recommended GWG	8 (28.6)	15.23 ± 1.10	0.39 ± 0.05
Above recommended GWG	0.0 (0.0)	Not applicable	Not applicable
Normal weight (18.5–24.9)			
Less than recommended GWG	98 (68.5)	9.31 ± 1.32	0.27 ± 0.06
Recommended GWG	43 (30.1)	13.06 ± 1.34	0.35 ± 0.08
Above recommended GWG	2 (1.4)	17.03 ± 0.78	0.46 ± 0.08
Overweight (25.0–29.9)			
Less than recommended GWG	2 (9.5)	6.60 ± 0.00	0.23 ± 0.03
Recommended GWG	15 (71.4)	9.15 ± 1.20	0.26 ± 0.08
Above recommended GWG	4 (19.0)	13.73 ± 1.53	0.40 ± 0.09
Overall			
Less than recommended GWG	120 (62.5)	9.34 ± 1.44	0.23 ± 0.03
Recommended GWG	66 (34.4)	12.43 ± 2.30	0.34 ± 0.10
Above recommended GWG	6 (3.1)	14.83 ± 2.12	0.38 ± 0.07

*BMI* Body mass index, *GWG* Gestational weight gain, *Sd* Standard deviation, *IOM* Institute of Medicine, *MRGWG* mean rate of gestational weight gain during the second and third trimesters

Table 5 shows participants mean rates of GWG during second and third trimesters against the Uganda MoH recommendation by BMI categories. The mean rate of GWG for underweight participants was 0.32 kg/week and this was lower than 0.51 which is recommended by the Uganda (MoH) and significantly different (95 CI: (− 0.22, − 0.15; *P* < 0.001). Similarly, the mean rate of GWG for normal weight participants was 0.30 kg/week and was significantly lower than 0.42 kg/week which is recommended by the IOM (95 CI: (− 0.13, − 0.11; *P* < 0.001). However, there was no statistically significant difference between the mean rate of GWG for overweight participants (0.28 kg/week) and that recommended by the Uganda MoH (95 CI: (− 0.04, − 0.05; *P* = 0.869).

**Table 5:** Comparison of participants’ mean rates of GWG by BMI category against MoH recommendation

Pre-pregnancy BMI (kg/m^2^)	Mean GWG (kg/week)	Test value^a^	MD (kg/week)	*P* value (95% CI)
Underweight (< 18.5)	0.32	0.51	−0.19	< 0.001 (−0.22, −0.15)
Normal weight (18.5–24.9)	0.30	0.42	−0.12	< 0.001 (− 0.13, − 0.11)
Overweight (25.0–29.9)	0.28	0.28	0.00	0.869 (− 0.04, 0.05)
Obese (≥ 30.0)	None	0.22	None	None

^a^Test value is the rate of gestational weigh gain recommended by the IOM for BMI category, *GWG* Gestational weight gain, *MD* mean difference between the participants’ mean and that recommended by the Uganda MoH, *CI* confidence interval

## Discussion

Generally, it is recognized that the pattern of maternal GWG has significant influence on fetal growth [18]. Although, several studies have reported how differences in the timing of maternal weight gain may be related to fetal growth outcomes [7, 19], the information on the pattern of GWG by pre-pregnancy BMI in Uganda is very limited.

The pre-pregnancy BMI of the participants was close to what was reported in Nigeria [10] but was lower than what was reported in another study in Nigeria and also in Brazil [20, 21]. Our low BMI could be explained by the fact that our study enrolled only women who were pregnant for the first or second time and the majority were relatively young, mean age of 20.9 years. This is in agreement with studies which have reported that BMI increases with increasing parity and age [22]. Furthermore, our study found about three quarters of participants with a normal pre-pregnancy BMI. This is close to that reported in the Nigerian study [10], but is higher than the 65.4% that was reported in a study conducted in Vietnam [23].

The mean GWG among pregnant women who were overweight before pregnancy was lower compared to those who were underweight and normal weight (Table 4). This finding is similar to results from previous studies done in both developed and developing countries [12, 19]. This is partially explained by the lower recommended gestational weight gain for overweight women as compared to those with normal BMI [18]. Although the mean GWG was lower among overweight participants in this study, it was the BMI category where we registered the highest percentage of participants gaining adequate (71.4%). Furthermore, it was only in the overweight category where we found participants gaining excessive gestational weight. This data suggests that women who enter pregnancy when overweight have reduced chances of getting inadequate gestational weight gain as compared to those with normal BMI or underweight. Our findings are in agreement with the findings of other studies done in Brazil and England [24, 25]. It is also important to note that the mean rates of GWG for underweight and normal weight participants during second and third trimester were lower and significantly different from those recommended by the Uganda MoH. However, there was no difference between the mean rate of GWG for overweight participants and that recommended by the Uganda MoH. We had 1.4% and 19.0% of our participants gaining excessive gestational weight in the normal weight and overweight categories respectively (Table 5). As much as we did not have obese (BMI ≥ 30 kg/m^2^), there is evidence to show that overweight and obese women have a higher risk of gaining excessive gestational weight [24, 25].

Overall the proportion of women who gained less the needed weight during pregnancy was 62.5%. This is close to with the findings of Farhana et al. (2015) in rural Malaysia [11]. This closeness in findings could be because the two study populations had education level of secondary and above and achieved the required number of antenatal clinic visits. Our finding is higher than that of other studies conducted in other developing countries like countries Malaysia and Iran and developed countries like United States of American and Canada [13, 19]. Norfazlin et al., (2012) found inadequate gestational weight gain in 42.9% of women in an urban setting in Malaysia [19]. Our higher proportion of women who gained less the needed weight could be as a result of poverty, food insecurity, economic instabilities and frequent infections which are common in Sub Sarahan Africa [26]. However, our proportion of women gaining inadequate gestational weight is lower than 97% and 80% that have been reported among pregnant women in Nigeria in 2014 and Iran in 2005 respectively [10, 27]. The high percent in Nigerian was attributed to dietary, genetic and environmental factors while in Iran, it was attributed to illiteracy. Furthermore, our study found a low prevalence (34.4%) of participants gaining adequate weight based on their pre-pregnancy BMI according to the recommendation of the Uganda MoH [4]. This is close to 32.5% that was found by Farhana et al.*,* (2015) in their study on Malaysian pregnant women in the rural area [11]. However, our finding of is lower than the 27.5% and the 27.7% that were reported in urban Brazil and urban Malaysia respectively [24, 25]. Although our prevalence of women gaining adequate gestational weight is low, it is higher than the 3.1% that was reported in Nigeria [10]. The majority of our participants with underweight and normal BMI gained less than the recommended weight gain for their pre pregnancy BMI whereas most of the overweight participants gained weight within the acceptable ranges. In addition, only a few women in the normal weight and overweight categories gained weights higher than the recommended. These findings are similar to the findings of Esimai and Ojofeitimi (2014) in Nigeria [10]. However, the findings of our study are different from those in the study done in Iran which registered the highest percentage of participants gaining inadequate gestational weight in the overweight category [28].

Despite the majority of our population having normal (healthy) BMI at the time of getting pregnant and existence of guidelines on maternal nutrition in Uganda, only 34.4% of our participants gained adequate gestational weight. This shows that, in Uganda, prenatal care services are inadequately addressing maternal weight gain during pregnancy. This may be true since reports in Uganda indicate that there is shortage of trained and motivated health care professionals including midwives who are central to the provision of antenatal services [4, 5]. Furthermore, less than 50% of the Ugandan women attend four quality antenatal visits [5]. Besides in other developing countries it has been observed that more than 50% of the women who come for antenatal care are not counseled on specific weight gain for their pre-pregnancy BMI [16]. This means that the majority of pregnant women in Uganda do have minimal access to interventions that address maternal malnutrition since this is part of the antenatal care package [4]. Unfortunately, this study did not collect data on number of antenatal visits. However, existing data show that several factors are responsible for maternal health outcomes in Sub Saharan Africa [29, 30].

The strength of our study includes the prospective design which followed women after inclusion until delivery. We excluded women with severe co-morbidities that could influence our results, thus enrolling a more uniform population for which we can generalize our finding to a similar population. However, we did not collect data on other risk factors for inadequate GWG such as level of physical activity, anemia, number of antenatal visits, and previous poor pregnancy outcome for those pregnant for the second time which could occur in Uganda. In addition, this study did not take into account other infections, such as malaria and helminths, which are prevalent in Uganda and have been associated with inadequate GWG [31, 32] neither did we collect data on whether the women received counseling on optimal GWG.

## Conclusion

Despite the availability of guidelines to ensure adequate GWG in Uganda, 62.5% of the women pregnant for the first or second time in low income urban Kampala do not gain adequate gestational weight. However, women who are overweight prior to getting pregnant have higher chances of gaining adequate gestational weight. We recommend that studies be carried out to assess whether the Uganda MoH recommendations for weight gain during are appropriate for preventing adverse pregnancy outcomes across populations in Uganda.

## Abbreviations

BMI: Body mass index
GW: Gestational weight
GWG: Gestational weight gain
IOM: Institute of Medicine
Kg: Kilograms
M: Metres
MoH: Ministry of Health
Sd: Standard deviation
